# Human nasal wash RNA-Seq reveals distinct cell-specific innate immune responses in influenza versus SARS-CoV-2

**DOI:** 10.1172/jci.insight.152288

**Published:** 2021-11-22

**Authors:** Kevin M. Gao, Alan G. Derr, Zhiru Guo, Kerstin Nündel, Ann Marshak-Rothstein, Robert W. Finberg, Jennifer P. Wang

**Affiliations:** 1 Department of Medicine and; 2 Department of Bioinformatics and Integrative Biology, University of Massachusetts Chan Medical School, Worcester, Massachusetts, USA.

**Keywords:** COVID-19, Infectious disease, Influenza, Innate immunity

## Abstract

**BACKGROUND:**

Influenza A virus (IAV) and SARS-CoV-2 are pandemic viruses causing millions of deaths, yet their clinical manifestations are distinctly different.

**METHODS:**

With the hypothesis that upper airway immune and epithelial cell responses are also distinct, we performed single-cell RNA sequencing (scRNA-Seq) on nasal wash cells freshly collected from adults with either acute COVID-19 or influenza or from healthy controls. We focused on major cell types and subtypes in a subset of donor samples.

**Results:**

Nasal wash cells were enriched for macrophages and neutrophils for both individuals with influenza and those with COVID-19 compared with healthy controls. Hillock-like epithelial cells, M2-like macrophages, and age-dependent B cells were enriched in COVID-19 samples. A global decrease in IFN-associated transcripts in neutrophils, macrophages, and epithelial cells was apparent in COVID-19 samples compared with influenza samples. The innate immune response to SARS-CoV-2 appears to be maintained in macrophages, despite evidence for limited epithelial cell immune sensing. Cell-to-cell interaction analyses revealed a decrease in epithelial cell interactions in COVID-19 and highlighted differences in macrophage-macrophage interactions for COVID-19 and influenza.

**Conclusions:**

Our study demonstrates that scRNA-Seq can define host and viral transcriptional activity at the site of infection and reveal distinct local epithelial and immune cell responses for COVID-19 and influenza that may contribute to their divergent disease courses.

**Funding:**

Massachusetts Consortium on Pathogen Readiness, the Mathers Foundation, and the Department of Defense (W81XWH2110029) “COVID-19 Expansion for AIRe Program.”

## Introduction

Influenza A virus (IAV) is a myxovirus that causes yearly epidemics (except in 2020) and has caused multiple pandemics in the past. Infection with IAV is characterized by the onset of fever, chills, and myalgias and may be complicated by viral pneumonia. Fatal disease is often associated with secondary bacterial infection, particularly in the elderly. In contrast, severe acute respiratory syndrome coronavirus 2 (SARS-CoV-2) is a novel coronavirus that emerged in 2019 and spread rapidly around the world, causing the coronavirus disease 2019 (COVID-19) pandemic. COVID-19 is a multifaceted disease with diverse manifestations, ranging from asymptomatic infection to severe illness requiring hospitalization or intensive care unit management with high morbidity and mortality (1, 2). Diabetes, advanced age, cardiovascular disease, chronic lung disease, hypertension, and obesity are all risk factors for the progression and poor prognosis during COVID-19 (3–7).

IAV enters cells via sialic acid found on cell surfaces throughout the body, whereas SARS-CoV-2 cellular entry is mediated by the cellular receptor angiotensin-converting enzyme 2 (ACE2) and the transmembrane protease, serine 2 (TMPRSS2). Each is expressed in the human respiratory tract, the predominant site of human infection with these viruses. The increased incidence of morbidity and mortality associated with SARS-CoV-2 compared with IAV is thought to result from specific host immune responses and the type and extent of tissue injury caused by the virus (8). Virus-driven hyperinflammation contributes to the severe disease manifestations of COVID-19, with heightened proinflammatory cytokine and chemokine responses contributing to the cytokine storm and corresponding with severe disease (9). Immunologic studies of samples from patients with acute COVID-19 performed at the single-cell level in peripheral blood mononuclear cells (10–16) and in the lower respiratory tract (17–19) provide insight toward understanding hyperinflammation. Yet a study comparing lower airway and peripheral blood samples from patients with severe disease reported no major differences in cell populations obtained via endotracheal tube washes from intubated patients with COVID-19 versus controls without COVID-19 (20). Tracheal samples from severely ill patients with COVID-19 were characterized by a predominance of myeloid lineage–derived cells, with few T cells and negligible numbers of B cells and innate lymphoid cells (21–23).

To better understand differences between influenza and COVID-19, we compared host cellular responses in the upper respiratory tract (i.e., the primary site of infection) for both infections. We applied single-cell RNA sequencing (scRNA-Seq), specifically a microwell-based scRNA-Seq technology (24), to nasal wash samples collected from adult donors presenting at our medical center with acute SARS-CoV-2 infection or acute influenza. We characterized diverse cell types participating in the first line of defense against each viral infection, defining cell-type distribution and differential host transcriptional responses. Our study provides insight into interactions between epithelial cells and immune cells responding to SARS-CoV-2 infection and those responding to IAV in the human nasal tract.

## Results

### Single-cell transcriptional landscape of human nasal wash cells from donors with acute COVID-19 or influenza.

**Figure 1:**
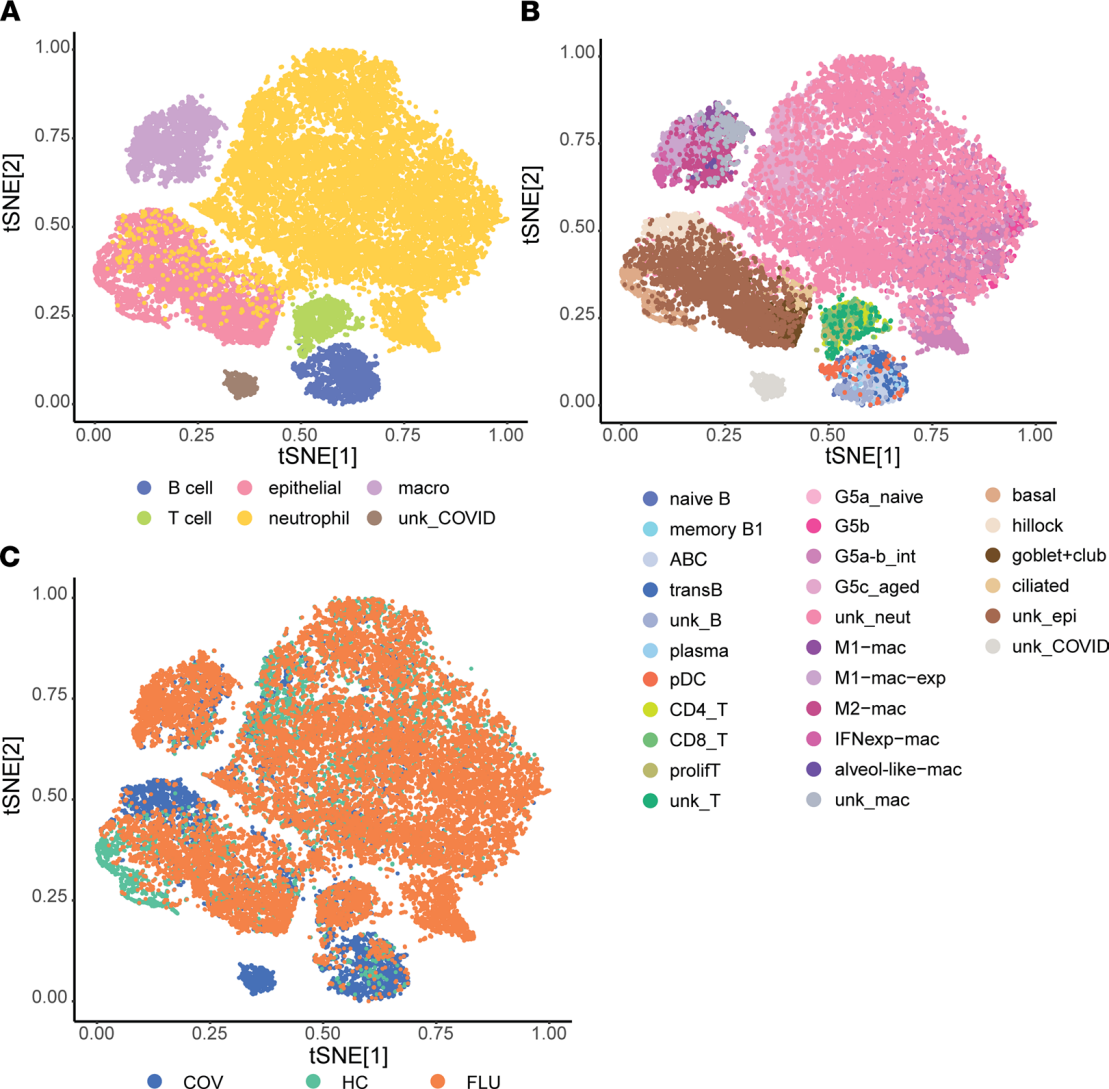
scRNA-Seq analysis reveals specific cell populations in nasal wash samples from patients with COVID-19 and influenza as well as healthy controls. Seq-Well data were analyzed using t-distributed stochastic neighbor embedding (tSNE) mapping and unsupervised density clustering. The cell types were further classified into subtypes on the basis of clustering and high expression of specific markers to obtain a visual map of distinct cell clusters within major cell types (**A**) and subtypes (**B**). ABC, age-dependent B cells; alveol-like, alveolar-like; G5a_naive, naive neutrophils; G5b, IFN-experienced neutrophils; G5a-b_int, intermediate neutrophils; IFNexp, IFN-experienced; G5c_aged, aged neutrophils; M1-mac, M1-like macrophages; M1-mac-exp, M1-like IFN-experienced macrophages; M2-mac, M2-like macrophages; macro, macrophages; pDC, plasmacytoid dendritic cells; prolifT, proliferative T cells; transB, transitional B cell; unk, unknown. (**C**) Distribution of cells by donor disease state is shown. COV, COVID-19; HC, healthy control; FLU, influenza.

From January to June 2020, we collected nasal wash samples from adults who presented to our medical center and were diagnosed with SARS-CoV-2 infection by PCR (*n* = 8, 7 donors) as well as samples from adults diagnosed with IAV by PCR (*n* = 14, 8 donors). We used nasal wash samples from healthy adult volunteers without evidence of respiratory viral infection as a comparator (*n* = 6, 6 donors). Additional details on the donors (e.g., duration of symptoms, age, sex, disease severity) are available in Supplemental Data File 1 (supplemental material available online with this article; https://doi.org/10.1172/jci.insight.152288DS1). scRNA-Seq libraries were generated using the Seq-Well (Seq-Well S^3) platform (25) and then sequenced on the Illumina NextSeq 500. Samples were filtered to remove cells with < 500 transcripts/cell (i.e., too few transcripts for analysis) and >33% mitochondrial transcripts, which are indicative of dead or dying cells (26, 27). We obtained a total of 29,406 cells across all samples, specifically 5669 cells from donors with COVID-19, 5629 cells from healthy donors, and 18,108 cells from IAV-positive donors. We found a total of 46,785,109 transcripts with an average of 1,591 transcripts per cell and an average of 703 genes detected per cell (Supplemental Data File 2). To distinguish specific nasal cell populations, we performed t-distributed stochastic neighbor embedding (tSNE) mapping and unsupervised density clustering and obtained distinct clusters (Figure 1A). These were composed of epithelial cells and immune cells, including neutrophils, macrophages, T cells, and B cells, based on the presence of cell-specific markers (Table 1). Cells were further classified into subtypes based on clustering and high expression of specific markers (Figure 1B, Supplemental Figure 2, and Table 1). Examples of specific transcriptional marker expression displayed in cell types are available in Supplemental Figure 1, and Figure 1C shows the distribution of cells by disease state. Epithelial cells and neutrophils accounted for the largest number of cells in all 3 groups (healthy, COVID-19, and influenza).

### Effect of disease state on cell types in the nasopharynx.

Because COVID-19 frequently has a prolonged disease course compared with influenza and the time from symptom onset to sample collection could affect the cell populations in the upper respiratory tract, we focused on specific samples collected from donors (COVID-19, *n* = 5; healthy, *n* = 6; influenza, *n* = 5; additional details in Supplemental Data File 1). To mitigate for timing of sample collection (i.e., early in influenza, late in COVID-19), we included only donor samples collected at least 4 days but no more than 15 days from onset of symptoms. Because numbers of cells sequenced from each sample were not equal, certain donors contributed disproportionately within specific cell-type lineages. To minimize sampling bias and skewing, we determined the number of cells contributed by each donor to each cell lineage type. We then calculated the median cell count across all donors for a particular cell lineage type and randomly sampled at most this median value of cells from each donor for downstream differential gene expression analysis (see Supplemental Data File 1). This resulted in the number of cells analyzed for each disease state being more consistent (2063 cells from donors with COVID-19; 2581 cells from healthy donors; 5631 cells from donors with IAV) while maximizing the total number of cells assessed in our analysis. Hence, the final “balanced” analysis was normalized for both symptom duration and cell counts obtained from each patient.

### Effect of disease state on epithelial cell subsets.

**Figure 2:**
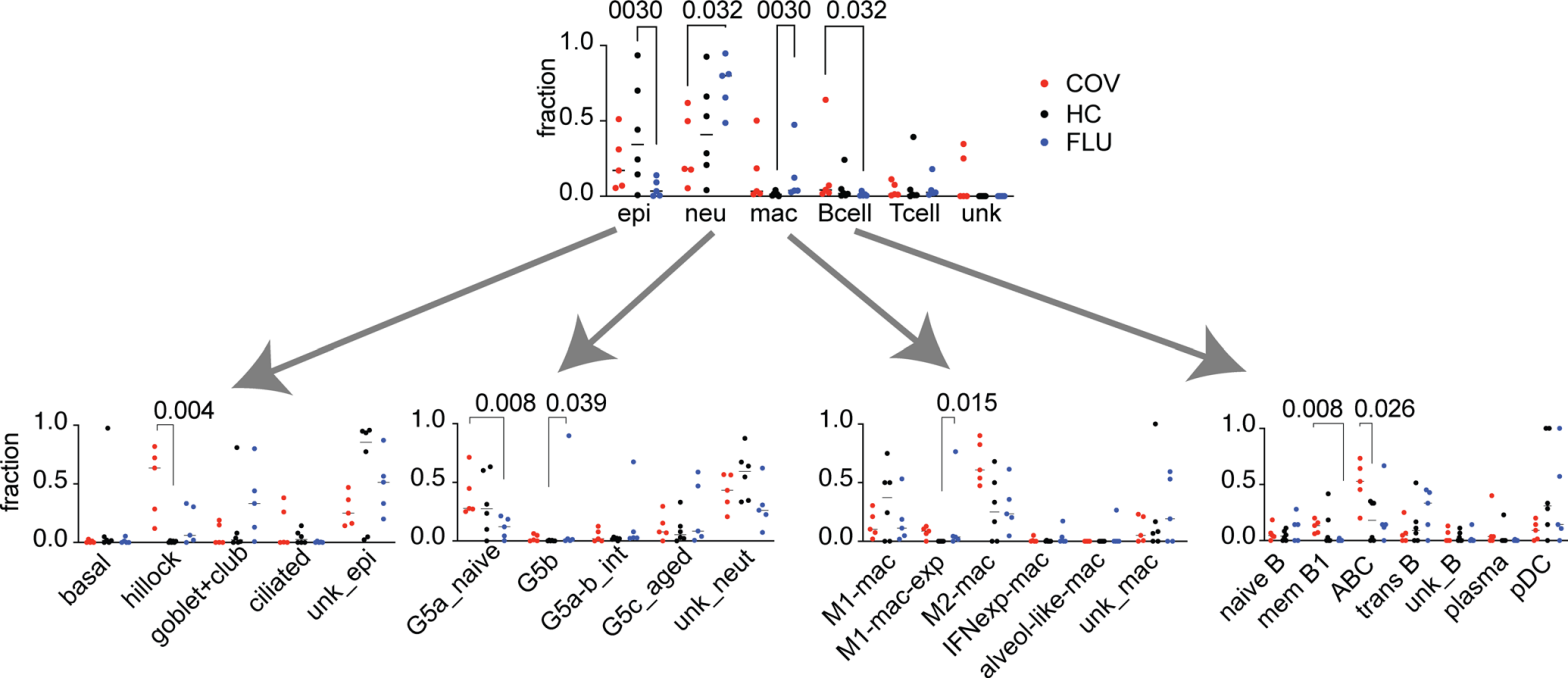
scRNA-Seq analysis identifies shifts in proportions of specific cell types in nasal washes from patients with COVID-19 and those with influenza compared with healthy controls. Fractions of major cell types identified from samples from patients with COVID-19 (COV) and patients with influenza (FLU) compared with healthy controls (HC) (*n* = 5 for COV, *n* = 6 for HC, and *n* = 5 for FLU). Fractions of epithelial cell, neutrophil, macrophage, and B cell subtypes are shown separately. The multiple Mann-Whitney test (unpaired, nonparametric with multiple comparisons) was used to determine *P* values. ABC, age-dependent B cells; alveol-like, alveolar-like; G5a_naive, naive neutrophils; G5b, IFN-experienced neutrophils; G5a-b_int, intermediate neutrophils; G5c_aged, aged neutrophils; IFNexp, IFN experienced; M1-mac, M1-like macrophages; M1-mac-exp, M1-like IFN-experienced macrophages; M2-mac, M2-like macrophages; mac, macrophages; neut, neutrophil; pDC, plasmacytoid dendritic cells.

Samples from donors with COVID-19 or influenza had a reduced proportion of basal epithelial cells compared with samples from healthy controls, while COVID-19 was associated with a profound increase in the number of hillock-like epithelial cells (Figure 2). To identify specific transcripts that were affected, we used Gene Ontology (GO) analysis to gain insight into the differential expression (DE) within cell subsets (Supplemental Data File 3) for major cell types (Supplemental Data File 4). Using a minimum 1.0 log_2_ fold change (2×) increase or decrease as a threshold, we found distinct sets of transcripts that were relatively increased or decreased as a function of disease state. GO analysis for epithelial cell transcripts comparing donors with influenza to healthy controls revealed an increase in transcripts related to type I IFN signaling and a decrease in those related to keratinization, differentiation, and epidermis development (Table 2). Expression of transcripts related to the type I IFN signaling pathway and IFN-γ–mediated signaling pathway and the response to IFN-β were all decreased in samples from donors with COVID-19 compared with those from donors with influenza. Transcripts related to keratinocyte differentiation and keratinization were decreased both in donors with influenza and donors with COVID-19 as compared with healthy controls.

### Effect of disease state on immune cell subsets.

Here, we focused on neutrophils, macrophages, and B cells, given the differences observed in these populations based on disease state. Influenza samples had a higher proportion of neutrophils compared with samples from healthy controls or COVID-19 samples (Figure 2). Exogenous IFN has important effects on neutrophils and activates JAK and STAT pathways resulting in the transcription of multiple genes. Neutrophils can be classified as IFN experienced based on their expression of IFN-stimulated genes (ISGs), such as IFN-induced GTP-binding protein Mx1 (*MX1*) and IFN-induced protein with tetratricopeptide repeats 2 (*IFIT2*). IFN-experienced neutrophils were more frequently found in samples from patients with acute influenza compared with patients with COVID-19, consistent with SARS-CoV-2’s ability to shut down IFN responses.

Macrophages can be subtyped by their phenotype and functional characteristics. Traditionally, M1 macrophages are IFN activated and cytotoxic for bacteria and tumor cells, whereas M2 macrophages are characterized by their association with tissue repair and antiinflammatory processes. More recent descriptions for macrophage states utilize cluster-specific markers and their relationship to monocytes (28). We assigned classifications of M1-like and M2-like macrophages in accordance with Liao et al. (19). Interestingly, the percentage of M1-like macrophages was increased in influenza samples, while the percentage of M2-like macrophages was substantially elevated in COVID-19 samples compared with influenza samples.

Age-dependent B cells (ABCs) were significantly enriched in COVID-19 samples following balancing (Figure 2). Because ABCs are traditionally associated with age, we sought to determine if donor age correlated with the proportion of B cells that were ABCs. COVID-19 donors were indeed overall older than influenza or healthy control donors in our study population (COVID-19, ages 58–82 years; influenza, ages 40–58 years; healthy controls, ages 35–63 years, see Supplemental Data File 1). Differences in the age ranges limit any further interpretation with subgroups. Of note, ABCs have been associated with production of autoantibodies in systemic lupus erythematosus (29). The high proportions of ABCs and plasma cells in patients with COVID-19 in our study mirrors reports of increases in these cell subsets in the peripheral blood of patients with severe COVID-19 (30, 31). Whether increases in ABCs might be responsible for increased frequency of autoantibodies in COVID-19 remains to be determined.

### Analysis of cell-cell lineage interactions.

**Figure 3:**
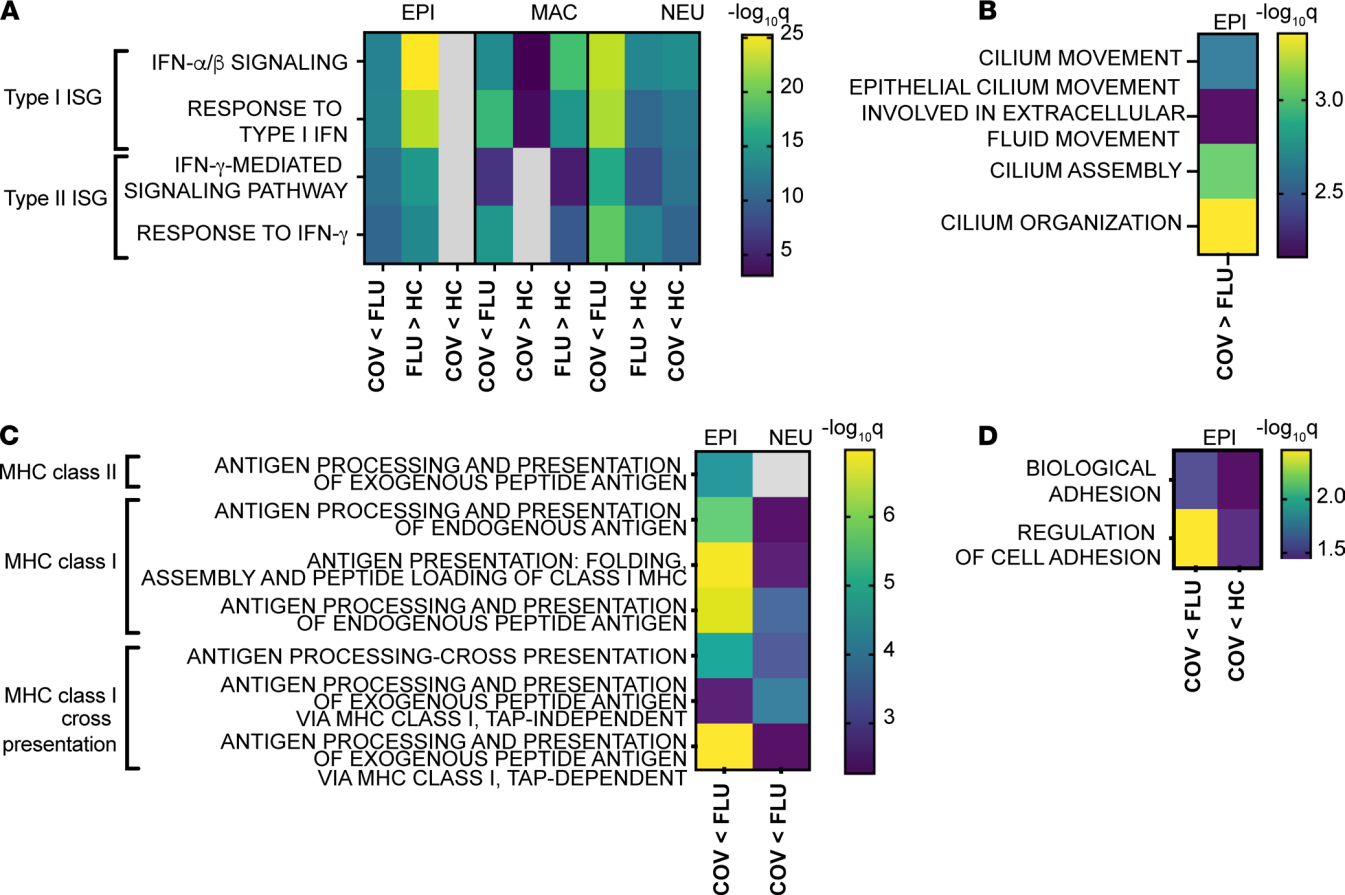
Pathway enrichment analysis of differentially expressed genes in nasal wash samples from COVID-19 donors, influenza donors, and healthy participants. Transcripts differentially increased (>) or decreased (<) for pairwise comparisons among COVID-19 (COV), influenza (FLU), and healthy control (HC) samples across cell types were identified. These transcripts were then used to identify statistically enriched GO terms. GO terms related to IFN signaling (**A**), ciliary function (**B**), antigen presentation (**C**), and cell-cell adhesion (**D**) are shown. In heatmaps, the color relates to the statistical significance of the enrichment, displayed as –log_10_*q*, where *q* is the adjusted *P* value. Gray shading indicates that statistical significance was not met. Each row represents an enriched GO term, while each column represents a cell type. EPI, epithelial; NEU, neutrophil; MAC, macrophage.

We performed a pathway enrichment analysis by generating gene enrichment maps on differentially expressed genes between the disease states and visualized these pathways as a network of enriched GO terms. We identified enriched GO annotations related to the IFN response and MHC class I antigen presentation in epithelial cells (Figure 3, A and C). Type I IFN response and MHC class I antigen presentation transcripts in neutrophils were decreased for COVID-19 samples compared with influenza samples (Figure 3, A and C). We also saw a loss of transcripts for MHC class I antigen presentation by epithelial cells in COVID-19 samples compared with influenza samples.

Analysis of transcripts from macrophages revealed an increase in type I IFN pathway genes from influenza samples compared with healthy control samples (Figure 3A and Supplemental Data File 4). Specifically, influenza samples maintained higher levels of type I IFN response–associated antiviral ISGs (e.g., *IFITM1*, *IFITM2*, *IFITM3*, *OAS2*, *OAS3*, *OASL*) and antigen presentation genes (e.g., *HLA-DRB5*, *HLA-C*, *B2M*, *HLA-B*, *HLA-E*) (see Supplemental Data File 4). In addition, despite an enrichment of typically regulatory M2-like macrophages in the COVID-19 nasal wash samples, a comparison of transcripts from M2-like macrophages revealed a dramatic increase in proinflammatory genes related to IL-1β and chemotaxis in COVID-19 samples compared with influenza samples (Table 3 and Supplemental Data File 3). Epithelia from COVID-19 samples showed enhancement of GO terms for ciliary function (Figure 3B) and a paucity of cell-cell type adhesion genes (Figure 3D). DE analyses are available for all cell types for the disease states and healthy controls (Supplemental Data Files 3 and 4).

### Analysis of receptor-ligand interactions.

**Figure 4:**
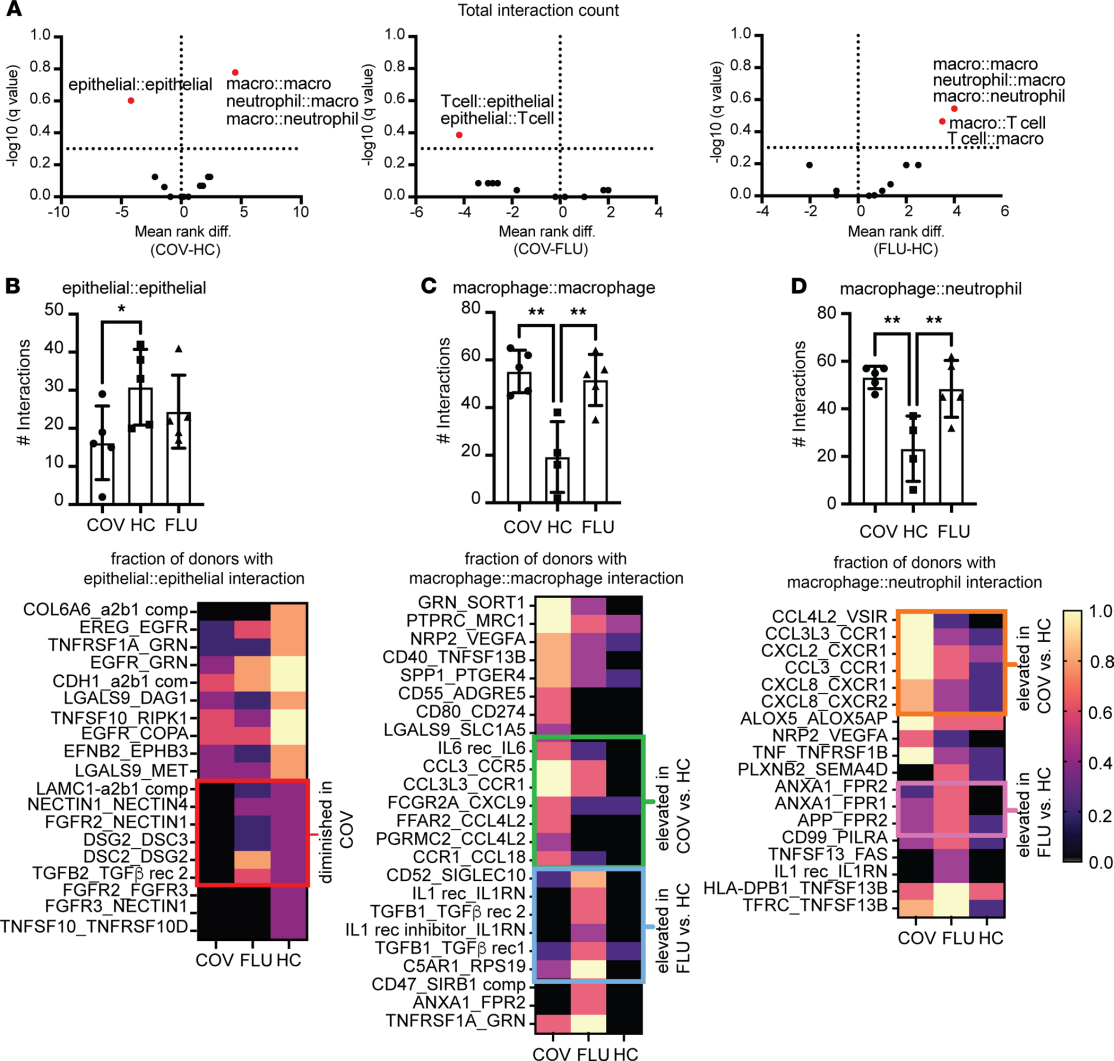
Epithelial cell–epithelial cell interactions are diminished while specific innate immune cell interactions are enhanced in COVID-19 compared with influenza. (**A**) Statistically significant receptor-ligand pair interactions between single cells across cell lineages were computed using CellPhoneDB. A multiple Mann-Whitney test was performed on cell lineage interactions for patients with COVID-19 (COV) and healthy controls (HCs), for COV and patients with influenza (FLU), and for FLU and HCs. Volcano plots show multiple statistical comparisons. Dots are annotated as a cell lineage pair *X*:*Y*, with *X* being the cell lineage expressing a receptor, and *Y* being the cell lineage expressing a ligand. The mean rank difference and adjusted *P* value are shown on the axes. (**B–D**) Significant receptor-ligand interactions are plotted across donor samples based on disease states. Composition and consistency of receptor-ligand interactions was assessed across disease states, with each heatmap row representing a receptor-ligand pair. (**B**) Epithelial cell–epithelial cell interactions diminished in frequency in COV vs. HC (differences ≥0.4). (**C**) Macrophage-macrophage interactions increased in COV vs. HC and in FLU vs. HC. (**D**) Macrophage-neutrophil interactions increased in COV vs. HC and in FLU vs. HC. Statistical significance for group-cell group interactions between patient samples across disease states (bar graphs) was determined by a Kruskal-Wallis test (*P < 0.05; **P < 0.01). Each heatmap column refers to the disease state, and the color represents the fraction of donors with statistical significance for annotated receptor-ligand interaction (CellPhoneDB analysis, *P* < 0.05). Note that for **C** and **D**, 1 HC donor sample did not have any macrophages or neutrophils, so interactions could not be reported. *n* = 5 for COV, *n* = 6 for HC, and *n* = 5 for FLU.

We performed receptor-ligand interaction analysis between lineages of cells for each sample using CellPhoneDB (see Supplemental Data File 5) to capture statistically significant interaction pairs between cell lineages across donor groups (Figure 4A). Influenza samples showed enhanced innate-innate (neutrophil and macrophage), innate-adaptive (macrophage and T cell), and adaptive-adaptive (T cells) interactions compared with healthy control samples. COVID-19 samples showed enhancement of innate-innate interactions but a strikingly reduced parenchymal-parenchymal (epithelial cells) interaction (Figure 4B). Additionally, we noted that adaptive-parenchymal interactions (epithelial and T cells) were diminished in COVID-19 samples compared with influenza samples.

To account for epithelial cell–epithelial cell interactions that were consistently diminished between COVID-19 samples and samples from healthy controls (Figure 4B), we determined which receptor-ligand interactions were consistently lost. To do this, we reported the fraction of patient samples having a particular receptor-ligand interaction (Figure 4B). We found that, while several interactions related to cell growth (EREG_EGFR; EGFR_COPA) and immune response (TNFRSF1A_GRN; TNFSF10_RIPK1) showed similar reductions in COVID-19 and influenza samples compared with healthy controls, COVID-19 samples showed a specific loss in interactions related to cell-cell adhesion, including formation of tight junctions and maintenance of barrier integrity (NECTIN1_NECTIN4, DSG2_DSC3, DSC2_DSG2) (Figure 4B, heatmap, red box).

While COVID-19 and influenza samples each showed significant increases in macrophage-macrophage interactions as compared with healthy control samples (Figure 4C), the specific interactions comprising this enhancement differed. We noted an increase in IL-6 receptor and IL-6 cytokine expression contributing to macrophage-macrophage interactions that was more pronounced in COVID-19 samples than influenza samples (Figure 4C, green box). In contrast, influenza samples showed enhancement of several immune regulatory interactions between macrophages that were absent or diminished in COVID-19 samples (Figure 4C, blue box). We also observed enhanced macrophage-neutrophil interactions in COVID-19 and influenza samples compared with healthy controls (Figure 4D), with specific differences in chemoattractant pathways. While macrophages in COVID-19 samples expressed chemokines that primarily engage CC and CXC family chemokine receptors on neutrophils (Figure 4D, orange box), macrophages in influenza samples expressed ligands that engage formyl peptide receptors (FPR), *FPR1* and *FPR2* (Figure 4D, pink box).

### Expression of viral transcripts in nasal wash cells as a function of viral infection.

**Figure 5:**
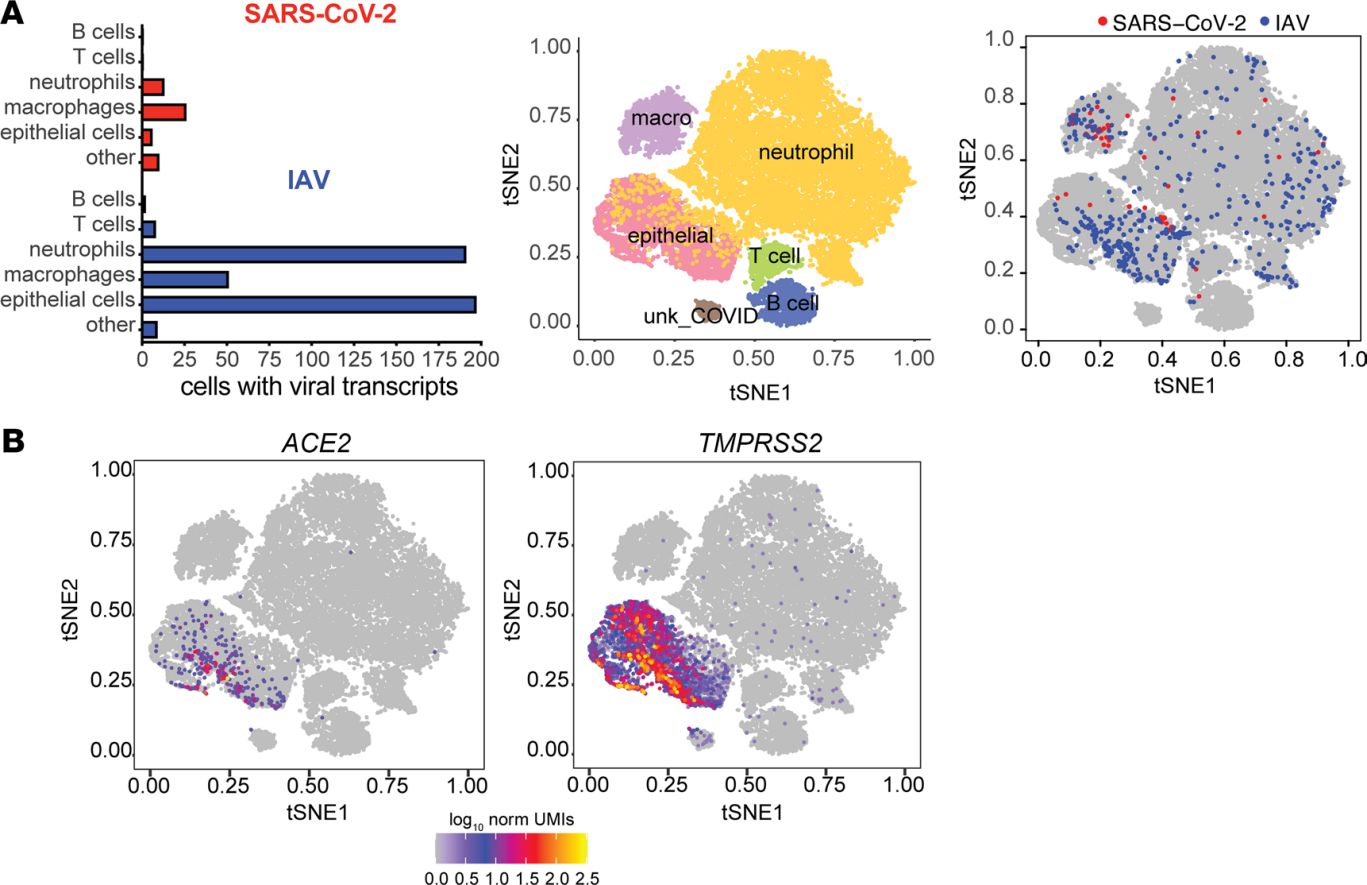
Cellular expression of viral transcripts and *ACE2* in nasal wash cells. (**A**) The distribution of SARS-CoV-2 and influenza A virus (IAV) transcripts by cell type, tSNE plot with major cell types identified, and tSNE plot showing cells with at least 1 viral transcript are displayed. (**B**) Expression of angiotensin-converting enzyme 2 (*ACE2*) and transmembrane protease, serine 2 (*TMPRSS2*) is limited to a subset of epithelial cells. macro, macrophages; unk, unknown.

Next, using all donor samples, we identified cells that contained either SARS-CoV-2 or IAV transcripts (see Methods). We found a total of 57 cells with SARS-CoV-2 transcripts and 458 cells with IAV transcripts distributed among various cell types (Figure 5A and Supplemental Data File 1). SARS-CoV-2 transcripts were found in only 4 COVID-19 donor samples, with the number of viral transcripts per cell ranging from 1 to 2060 (Supplemental Data File 2). We examined which cell types expressed *ACE2* and *TMPRSS2* and confirmed that the expression of each was limited to epithelial cells (Figure 5B). SARS-CoV-2 and IAV transcripts were found across a broad range of cell types (Figure 5A), including ciliated epithelial cells, a likely site of viral replication, as well as phagocytic cells (neutrophils and macrophages). Expression of viral transcripts in phagocytic cells likely reflects a waste pathway rather than evidence of active viral replication.

## Discussion

Using scRNA-Seq, we analyzed diverse human nasal cell populations that serve as the initial site of infection and first line of defense against SARS-CoV-2 and IAV. While numerous investigations examine lymphocytes and macrophages in the blood at the single-cell level, we focused on the site of the initial host encounter with the pathogen. Our study applied single-cell transcriptional analysis to define changes in the human nasal cell populations for two distinct upper respiratory virus infections. Using the nasal wash technique, we were able to harvest cells and process them immediately without freezing or delay. Epithelial cells, neutrophils, and some lymphocyte populations, particularly B cells, may not tolerate freezing and thawing, which may account for underrepresentation in other reports. Our ability to find diverse cell types is a testament to the fidelity of the technique.

Similar to a report by Deprez et al. (32), we noted the presence of “hillock”-like epithelial cells, originally described in the lower airway (33), in human nasal wash samples. Ziegler et al. identified these cells in patients with COVID-19 by sequencing frozen nasopharyngeal swabs (34). Interestingly, the percentage of hillock-like cells, which are associated with rapid turnover and immunomodulation (33), increased with both COVID-19 and influenza, but more so with COVID-19. Whether this finding relates to the rapid turnover of epithelial cells due to virus-mediated injury or is associated with cell migration warrants additional study. Likewise, a heightened frequency of ABCs in COVID-19 may drive disease-specific manifestations.

Our analysis of cell interactions also provides insight regarding differences in COVID-19 and influenza manifestations. We found that transcripts related to antigen presentation were diminished in epithelial cells and neutrophils from COVID-19 samples, which may contribute to the impaired recruitment of adaptive responses reported in patients with severe COVID-19 (35). We also determined that the ISG response to virus was lost in COVID-19 epithelia, suggesting a delay in viral sensing in the recruitment of an immune response.

As the upper respiratory tract is the entry site for both IAV and SARS-CoV-2, we were very interested in differences in cell distributions of viral transcripts. SARS-CoV-2 is only thought to enter and replicate in cells expressing ACE2. Sialic acid, the influenza receptor, is ubiquitously expressed, although most of the literature points to replication occurring predominantly in ciliated epithelial cells (36). We found influenza transcripts present in many different cell types; albeit, the largest number of transcripts were found in epithelial cells. In addition to epithelial cells, SARS-CoV-2 transcripts were found in macrophages and neutrophils, but it seems plausible that some of these transcripts were derived from ingested viral RNA. Previous single-cell studies of influenza in mouse lungs have noted the presence of virus in all cell populations studied (37). The SARS-CoV-2 and influenza transcripts seen in macrophages and neutrophils could result from phagocytic events and probably do not represent replication-competent transcripts.

In conclusion, this single-cell analysis of transcripts from nasal wash cells of healthy people or people infected with either SARS-CoV-2 or IAV allowed us to define differences in virus-host interactions for each disease and to consider how they might contribute to disease manifestations. Based on this study of the cells present in the upper respiratory tract, we hypothesize that the poor transcriptional response of epithelial cells to SARS-CoV-2, both in terms of IFN induction and antigen presentation, accounts for some of the differences in host responses to the two RNA viruses that may affect the disease course. Our findings confirm diminished IFN responses and dysregulatory patterns in macrophages and also bring attention to shifts in specific B cell populations in patients with COVID-19 that are not seen in patients with IAV. Such discoveries provide insight toward understanding the origins of the hyperinflammatory states and other complications all too frequently encountered with COVID-19.

## Methods

### Sample collection

#### Enrollment.

Nasal washes were obtained from healthy adult controls and from adults diagnosed with acute COVID-19 by PCR testing or IAV by rapid antigen test and/or by PCR testing. Samples were obtained by irrigation of each naris with up to 10 mL saline collected in a single container. The sample was then transported to the research laboratory for processing. Upon receipt, the sample was immediately stored on ice and 10 mL cell growth media (DMEM or RPMI1640 with 10% fetal bovine serum) was added. For viscous samples, freshly made cold PBS containing 10 mM dithiothreitol (Thermo Fisher Scientific, Pierce) was added to the sample at a 1:1 volume and incubated at room temperature with intermittent mixing until the sample liquified, and then an equal volume of 10% FBS cell growth media was added. The material was strained using a 40 μM nylon cell strainer (Corning) into a 50 mL centrifuge tube. Cells were pelleted at 200*g* for 10 minutes at 4°C. All but 1 mL supernatant was discarded, the pellet was resuspended in the remaining 1 mL supernatant, and the material was transferred to an Eppendorf tube and pelleted at 490*g* for 5 minutes. If the pellet contained visible blood, 200 μL RBC lysis solution (MilliporeSigma) was added to resuspend the pellet and incubated at room temperature for 2 minutes, after which 1 mL cell media was added, and the cells were pelleted at 490*g* for 5 minutes. The final pellet was resuspended in up to 1 mL of media and quantified.

### RNA sequencing

Seq-Well was used according to published methods (24) to capture single cells on a microwell array. Each microwell has only 1 bead carrying oligonucleotides that have a cell barcode, unique molecular identifiers (UMIs), and a polyT tail. Each array was loaded with 20,000 cells. Any remaining cells were put in TRIzol and stored at –80°C. After cell lysis, mRNA transcripts were captured by the oligonucleotides on the bead. The cDNA libraries were prepared using Illumina Nextera XT Library Prep Kits and sequenced using the Illumina NextSeq500.

### Computational analysis

#### Genome alignment and transcript quantification.

The Seq-Well paired-end fastq files contain the 12-base cell barcode and 8-base UMI in the R1 read and the 50-base transcript mRNA sequence in the R2 read. These paired-end reads were pre-processed using a custom Python script to extract the cell barcode and UMI from each R1 read and append them as a colon-delimited pair to the corresponding R2 read name. Reads with ‘N’s in either the cell barcode or UMI were discarded. The resulting fastq files were then processed through our DolphinNext analysis pipeline (38) as single-ended reads, removing reads from any cell barcode with fewer than 500 reads. The mRNA sequences were aligned to the human genome (hg38) using tophat2 (v2.0.12) with default settings and GENCODE (v28) transcript annotations. Gene transcripts were quantified using ESAT (https://github.com/garber-lab/ESAT; commit 22ac693c81839daf89609d6ae53dc141ff8a9d69), again using GENCODE (v28) transcript annotations. ESAT ignores reads that result from PCR duplication during the library preparation process using the UMI. If reads from the same cell barcode map to the same gene and have the same UMI, they are considered PCR duplicates, and only one is counted. The output of ESAT is an array containing the transcript counts for each gene for each cell.

#### Cell type identification.

Cell type identification was a multistep process using custom R scripts (R v3.5.0) based on the SignallingSingleCell package (https://github.com/garber-lab/SignallingSingleCell; commit 0f21359b1303839901cfd15927a0c91f0bf51f0c). All functions referenced refer to that package. The data for each sample were first processed to remove any cells with fewer than 500 transcripts, and cells with >33% of transcripts from mitochondrial genes, which indicate that the cell is dead or dying (26, 27). All samples were combined into a single ExpressionSet data object for further analysis (R/Bioconductor Biobase package). 2000 genes were selected using the variance-stabilizing transformation gene selection method to reduce noise introduced by low variance and low expression genes, followed by t-SNE mapping (dim_reduce() with default parameters) and density clustering (cluster_sc() with method=’density’ and num_clust=12) using the selected genes to produce an initial segmentation of the cells.

We then used the cluster and mapping boundaries to classify the cells into major groups using the expression levels of known marker genes to identify the groups. Clusters with high expression of *CD19* and *MS4A1* (CD20) were assigned to the B cell group, with high expression of *CD3G/D/E* were assigned to the T cell group, *FCGR3B* (CD16b) indicated neutrophils, *CD68* identified the macrophage group, and *KRT7* identified cells in the epithelial group.

After identifying the major cell groups, we remapped and clustered the cells in each group using the same steps as above, using separate 2000-gene sets selected for each group. To identify subtypes of cells within each group, we began by performing DE analysis, comparing the cells in each cluster to all other cells in that group, using the Bioconductor package edgeR. We then assigned specific cell types based on the most differentially expressed genes in each cluster combined with extensive literature searches. Because we depended on unsupervised mapping and clustering methods for the initial assignment of cells into groups, we occasionally identified cells in a group that needed to be reclassified. For example, we found plasma cells in both the T cell and B cell groups and neutrophils in the epithelial group.

#### DE analysis.

Finally, for each identified cell type, we selected the cells from each disease state (COVID-19, influenza, and healthy control) and performed DE analysis (edgeR) to identify genes that were most differentially expressed in each of the following conditions: COVID-19 versus healthy, influenza versus healthy, and COVID-19 versus influenza. If fewer than 10 cells were available for either condition, the DE analysis was not performed. In order to ensure that no single sample or disease duration had an undue influence on the DE results, we also selected a “balanced” set of cells for each major cell group from patients with COVID-19 and influenza with similar infection durations and an upper limit on the maximum number of cells from each patient.

#### Identifying cells with viral transcripts.

To identify cells containing viral transcripts, we constructed a BLASTN database containing the genomes of all SARS-CoV2 and influenza A genomes in the NCBI virus database (https://www.ncbi.nlm.nih.gov/labs/virus/). Using this database, we ran the NCBI magic-BLAST program on the single-ended transcript fastq files from all samples. We then processed the output through a custom Python script to label all matches to any influenza A genome as “flu’’ and all matches to any SARS-CoV-2 variant as “SARS-CoV-2,” and removed PCR duplicates using the UMIs as we described with the transcripts above. Finally, viral transcript counts were matched to the cell containing the transcript using the cell barcodes.

#### Pathway enrichment analysis.

Pathway enrichment analysis was performed on differentially expressed genes between disease states and visualized as a network of enriched GO terms. For GO enrichment analysis of unordered lists of significantly upregulated and downregulated genes, we used the online tool DAVID (https://david.ncifcrf.gov/) from National Institute of Allergy and Infectious Diseases/NIH. The GO enrichment analysis of ordered gene lists was performed using gProfiler gOST as an ordered query of significantly upregulated and downregulated genes using the databases “GO biologic process” and “REACTOME” (https://biit.cs.ut.ee/gprofiler/gost). Gene enrichment map files were obtained through this analysis and visualized by the Enrichment Map plugin in Cytoscape (http://apps.cytoscape.org/apps/enrichmentmap). Enriched GO annotations related to IFN response, antigen presentation, cell adhesion, and ciliary function were then identified for specific cell types.

#### Cell-cell interaction analysis.

Cell-cell interaction analysis was performed using CellPhoneDB to identify significant receptor-ligand pair interactions between cells annotated by their lineages on samples from each individual patient. The number of statistically significant intercell lineage interactions from each patient was then used to identify shifts in the number of interactions across patients between different disease states. For cell-cell lineages showing altered interaction counts across disease states, follow-up analysis of the quality of interactions was performed. This involved enumerating the significant receptor-ligand pair interactions for the cell-cell lineage pair of interest. The consistency of these receptor-ligand interactions was then measured as the fraction of patient samples in each disease state that were identified as showing the interaction as statistically significant within a particular cell-cell lineage pair.

#### Data availability.

The scRNA-Seq raw fastq files, gene by cell transcript counts matrix, and metadata file, including the donor ID, disease status, and viral transcript count for each cell, were deposited in the NCBI’s Gene Expression Omnibus database (GEO GSE176269).

### Statistics

Statistical tests were performed using GraphPad Prism version 9 and included the multiple unpaired, nonparametric Mann-Whitney test.

### Study approval

This study was reviewed and approved by the University of Massachusetts Chan Medical School’s Institutional Review Board (IRB protocol no. H00009277), and written informed consent was received from participants prior to inclusion in the study.

## Author contributions

JPW and RWF were involved in planning, supervised the work, and drafted the manuscript. ZG processed the samples for sequencing. AGD and KMG performed the bioinformatic analysis. KMG, AGD, KN, AMR, RWF, and JPW interpreted and discussed the results and commented on the manuscript. For the designation of co–first authorship, the method used to assign author order was based on a discussion between all authors.

## Supplementary Material

Supplemental data

ICMJE disclosure forms

Supplemental data set 1

Supplemental data set 2

Supplemental data set 3

Supplemental data set 4

Supplemental data set 5

## Figures and Tables

**Table 1:**
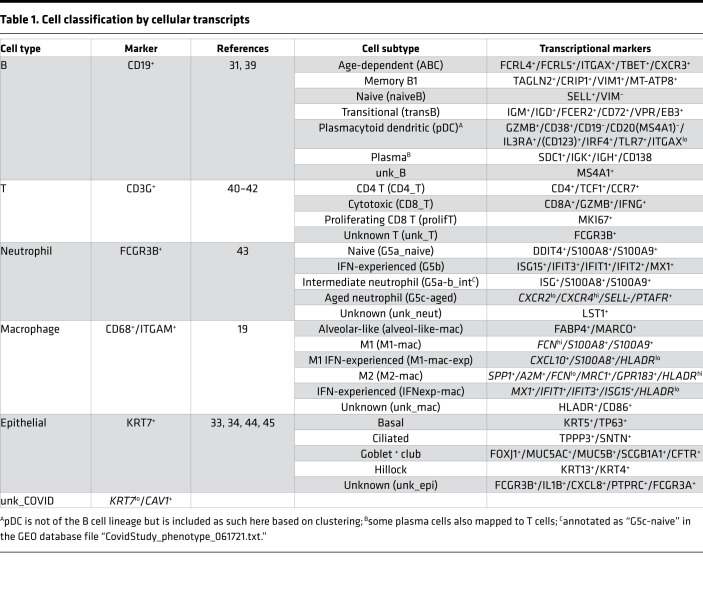
Cell classification by cellular transcripts

**Table 2:**
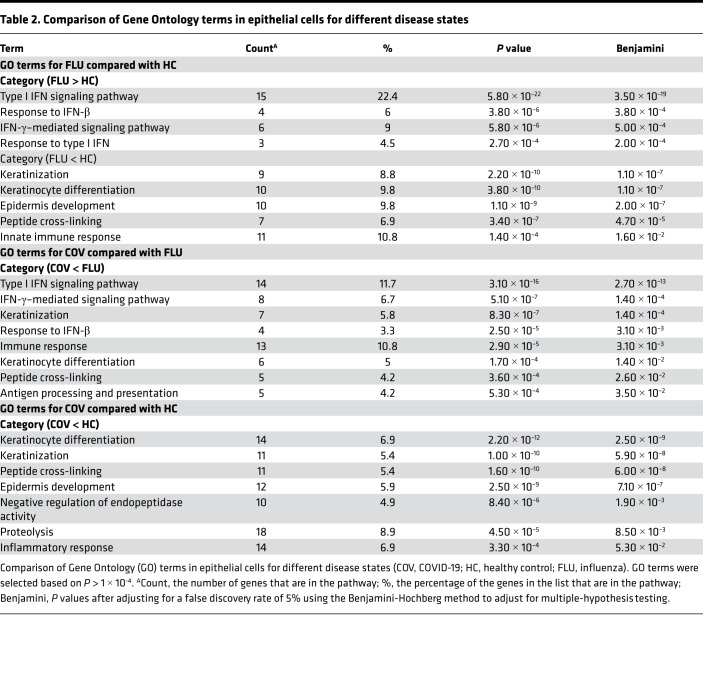
Comparison of Gene Ontology terms in epithelial cells for different disease states

**Table 3:**
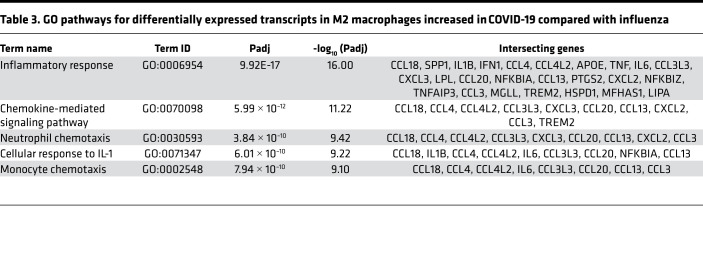
GO pathways for differentially expressed transcripts in M2 macrophages increased in COVID-19 compared with influenza

## Version 1 10/07/2021

In-Press Preview

## Version 2 11/22/2021

Electronic publication
