# Supplementary material for: Human nasal wash RNA-Seq reveals distinct cell-specific innate immune responses in influenza versus SARS-CoV-2
Source: JCI Insight. 2021 Nov 22;6(22):e152288. doi: 10.1172/jci.insight.152288 (PMC8663782; doi:10.1172/jci.insight.152288)
Supplement: Supplemental data [file jciinsight-6-152288-s095.pdf]

Supplemental Materials for:

Human nasal wash RNA-seq reveals distinct cell-specific innate immune responses between influenza and SARS-CoV-2

Kevin Gao[1*], Alan G. Derr[2*], Zhiru Guo[1*], Kerstin Nündel[1], Ann Marshak-Rothstein[1], Robert W. Finberg[1±], and Jennifer P. Wang[1±]

[1]Department of Medicine, University of Massachusetts Chan Medical School, Worcester, MA, USA

[2]Department of Bioinformatics and Integrative Biology, University of Massachusetts Chan Medical School, Worcester, MA, USA

*,[±] These authors contributed equally to the work, respectively.

Corresponding author:

Jennifer P. Wang, M.D.
Department of Medicine and Diabetes Center of Excellence
368 Plantation St.
ASC 7-2047
Worcester, MA, 01605, USA
Ph: 1-508-856-8414
email: Jennifer.Wang@umassmed.edu

Supplemental Figure 1. tSNE plots with specific transcriptional markers mapped.

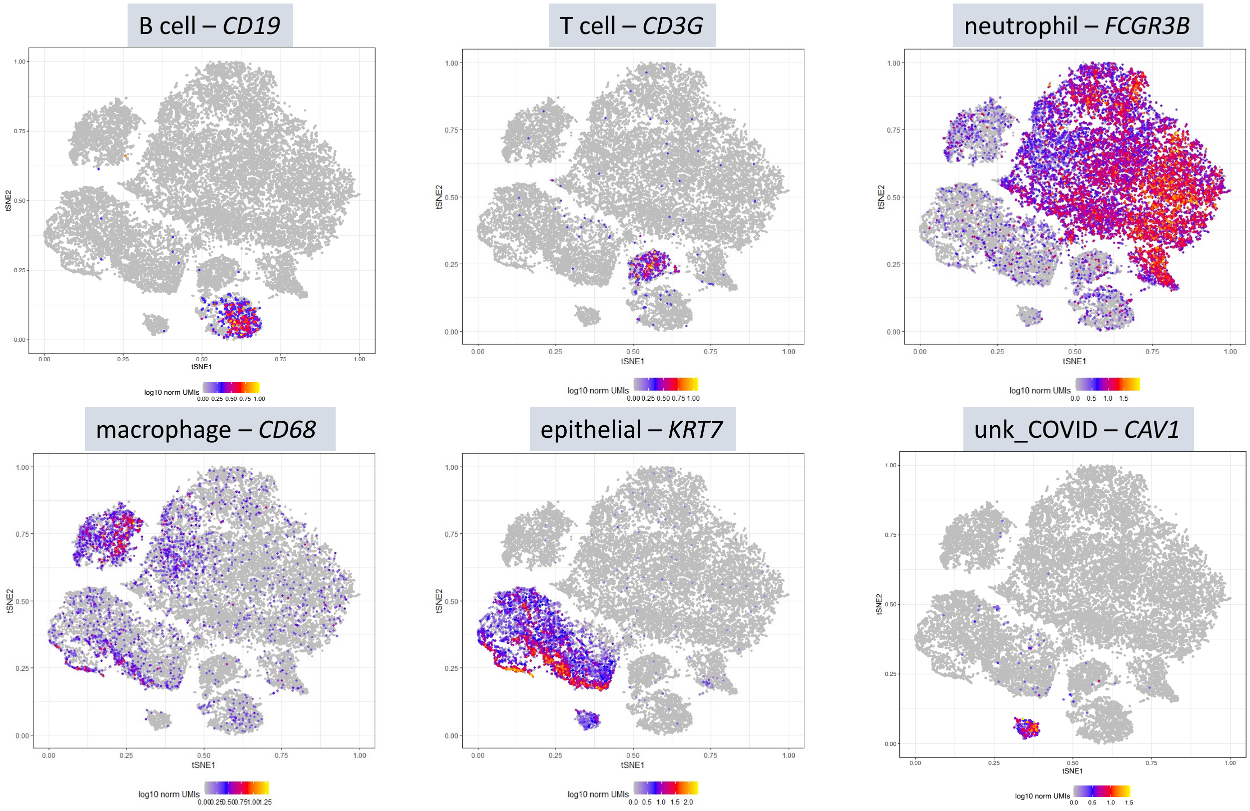

Note: We identified a unique population of cells expressing high levels of caveolin-1 (*CAV1)* and modest amounts of *KRT7* in two COVID-19 donor samples, labeled “unk_COVID” in **Figure 1**. Expression of *CAV1* has been described in both nasal and bronchial cells and is involved in cell proliferation and inflammation. CAV1 expression on epithelial cells could be a response to pulmonary injury.

Supplemental Figure 2. tSNE plots showing cell type distribution in donor samples from influenza, COVID-19, or healthy controls.

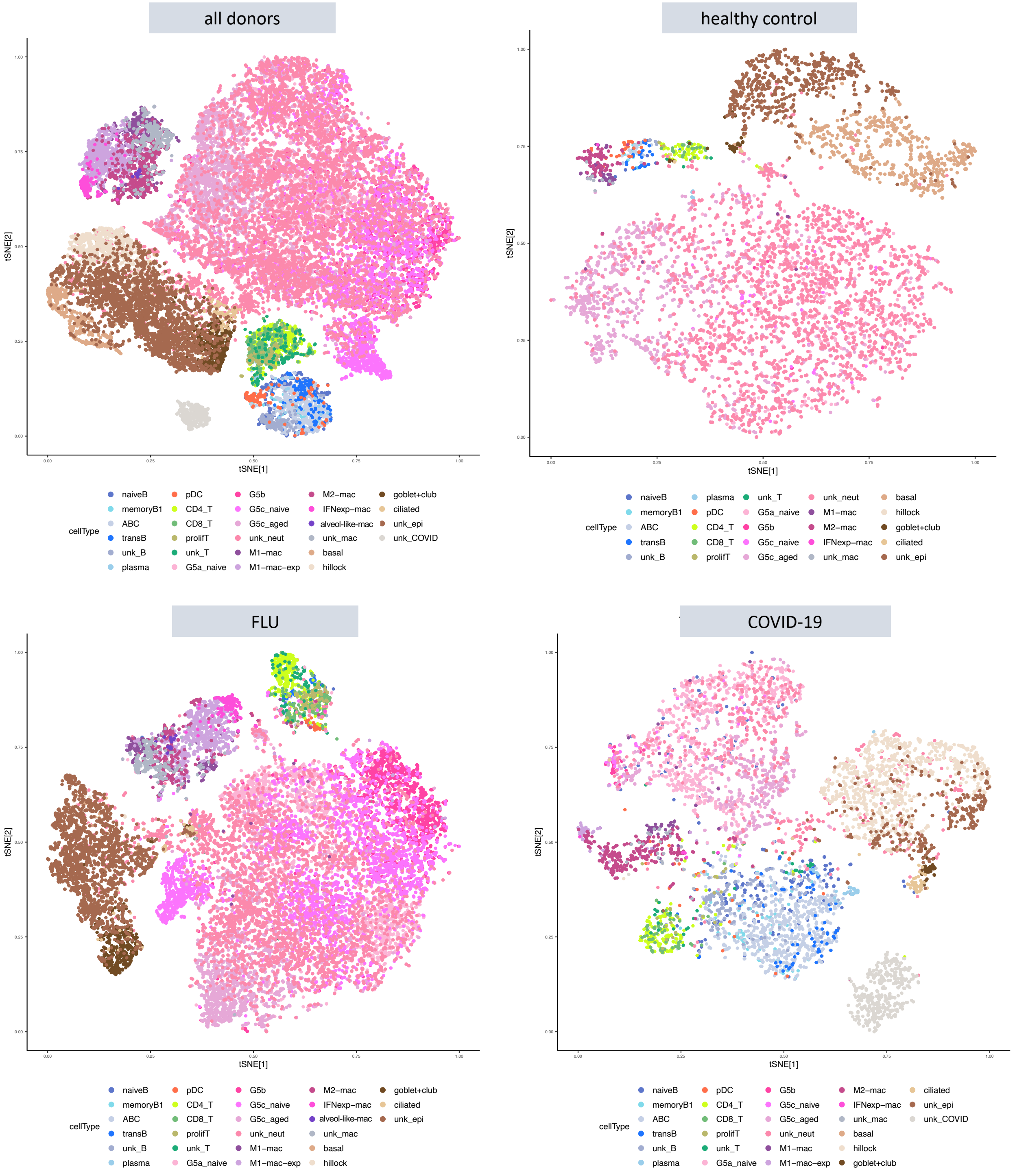

Please see supplementary excel files included with the manuscript:

Supplemental File 1. Donor status

Supplemental File 2. Cell type distribution

Supplemental File 3. DE analysis for cell subtypes, all samples

Supplemental File 4. DE and GO analysis for major cell types, balanced samples

Supplemental File 5. CellPhoneDB
